# Linking *Anopheles* bionomics and human behaviour in the Lao PDR

**DOI:** 10.1186/s12936-025-05435-1

**Published:** 2025-07-02

**Authors:** Sebastien Marcombe, Santi Maithaviphet, Rita Reyburn, Khamfong Kunlaya, Khambang Silavong, Bouasy Hongvanthong, Viengxay Vanisaveth, Viengphone Sengsavath, Vilasack Banouvong, Keobouphaphone Chindavongsa, Boualam Khamlome, Élodie A. Vajda, Edward Thomsen, Timothy Finn, Neil F. Lobo, Matthew Shortus

**Affiliations:** 1 WHO, World Health Organization, Vientiane, Lao PDR; 2 Center for Malariology, Parasitology and Entomology, Ministry of Health, Vientiane, Lao PDR; 3 Malaria Elimination Initiative, University of California, San Francisco, CA USA; 4 University of Notre Dame, Notre Dame, IN USA

**Keywords:** Lao PDR, Malaria, *Anopheles*, Vector, Bionomics, Entomology surveillance, Human behaviour observation

## Abstract

**Background:**

Laos aims to eliminate malaria by 2030, with residual transmission present in the south. Understanding how LLINs function in relation to vector and human behaviours, and linking this to epidemiological outcomes, is critical for improving vector control strategies. Integrating human behaviour observations (HBOs) with entomological and epidemiological data helps identify gaps in protection and inform national malaria programs.

**Methods:**

Entomological surveillance of *Anopheles* mosquitoes was conducted alongside HBOs in both rainy (July–September 2022) and dry (February–April 2023) seasons. Nine villages with high malaria burden were surveyed across three ecotypes: villages, cultivation sites, and forested areas. Mosquitoes collected via indoor and outdoor human landing catches (HLCs) were identified morphologically. HBOs recorded spatial and temporal human presence and LLIN use. Human biting rates (HBR) were adjusted with HBO data to quantify spatial and temporal protection and identify gaps in protection. *Anopheles* indoor resting rates were also assessed.

**Results:**

*Anopheles* mosquitoes (n = 1012) belonging to 12 species were collected, with the highest numbers from cultivation sites (n = 511), followed by villages (n = 302) and forests (n = 198). During the dry season, more *Anopheles* were found in cultivation sites than villages, while this was reversed in the rainy season. Human behaviour adjusted biting rates, or exposure was highest outdoors, particularly between 18:00–22:00 and 03:00–06:00 in villages. LLIN use in villages prevented 42% of total *Anopheles* bites and 83% of indoor bites during sleeping hours. However, LLINs only reduced exposure by 26% at cultivation sites, where *Anopheles* were primarily biting outdoors.

**Conclusions:**

This operational study demonstrates that peridomestic behaviours of both humans and *Anopheles* in Laos results in significant outdoor gaps in protection, especially in the early evening. High LLIN coverage remains essential for reducing indoor bites in villages. IRS may have limited impact due to the lack of indoor-resting vectors. Addressing these gaps requires innovative strategies. HBOs integrated with entomological data offer valuable insights into residual malaria transmission, particularly in outdoor and early evening settings, emphasizing the need for targeted interventions.

**Supplementary Information:**

The online version contains supplementary material available at 10.1186/s12936-025-05435-1.

## Background

Lao PDR (hereafter Laos) is targeting the elimination of *Plasmodium falciparum* by the end of 2025, and *Plasmodium vivax* by 2030, following a successful reduction in reported malaria cases from nearly 300,000 in 2000 [1] to 2130 in 2022 [2]. Health facility catchment areas and districts are classified into four strata based on risk, resulting in a tailored plan to intensify elimination activities in 121 out of 148 districts and accelerate burden reduction in the remaining 27 districts [3]. Currently, 95% of cases are concentrated in the five southern provinces (Savannakhet, Saravane, Champassak, Sekong, and Attapeu), with several focalized areas continuing to report high levels of *P. falciparum* despite full coverage of the core interventions, which include case management, community-based malaria workers, long-lasting insecticidal nets (LLINs), and active case detection through outbreak response activities [4].

Vector control relies primarily on LLINs, while indoor residual spraying (IRS) is employed as part of outbreak response activities in high-burden districts, contingent on the availability of financial and human resources. These efforts highlight the need for targeted strategies to address persistent transmission in focal areas, particularly in regions with high *P. falciparum* prevalence [4].

As Laos intensifies efforts to eliminate *P. falciparum*, the Center for Malariology, Parasitology and Entomology (CMPE) and the World Health Organization (WHO) have developed a target package of “accelerator strategies” to complement core interventions in the 19 highest-burden villages across three districts (Sepone and Nong Districts in Savannakhet Province, Boualapha District in Khammouane Province). Identified accelerator strategies include distribution of new LLINs (YorKool®), targeted distribution of long-lasting insecticidal hammock nets (LLIHNs) to forest goers, targeted drug administration, and intermittent preventive treatment for forest goers (IPTf), with planned scale-up of the activities across the target districts (started in March 2022). Accelerator strategies are targeted at both mobile and static high-risk populations (HRPs) across Laos, including forest goers, field (or agricultural) workers, ethnic minorities, and forest fringe populations.

To optimize the vector control components of the accelerator strategies, CMPE and its collaborators sought, in this operational and ministry-led study, to quantify the protection provided by existing measures and characterize the remaining gaps in protection. This exercise requires the comprehension of where and when vectors and humans overlap while factoring in presence and usage of interventions in place [5–12]. In Laos, no studies have been implemented using these methods but several have mentioned human behaviour indicators (sleeping with ITNs, house close to breeding site, sleeping away from home) as risk factors for malaria [10, 13–19].

## Methods

### Entomological surveillance planning tool (ESPT)

The ESPT (http://shrinkingthemalariamap.org/) is an operational decision-support tool designed to help malaria programs plan operational, question-based entomological surveillance activities for the collection of minimum essential indicators with limited funding envelopes towards providing representative data, thus supporting cost effective, locally tailored, and evidence-based vector control. In collaboration with WHO and the Malaria Elimination Initiative (MEI), CMPE applied the ESPT framework to the local context to enhance its current entomological surveillance and control strategies by integrating human behaviour observations (HBOs) with extended entomological collections (indoor resting catches, and 48-h collections in the deep forest). This ESPT-based entomological surveillance plan addressed the following priority program questions: (1) What are the proportions of exposure that LLINs, IRS, and LLIHNs can protect against among targeted high-risk populations (2) What are the remaining gaps in protection (that is, where and when is transmission still occurring and other interventions may be needed)? and, (3) How can existing interventions be optimized based on identified gaps in protection?

### Locations

**Fig. 1:**
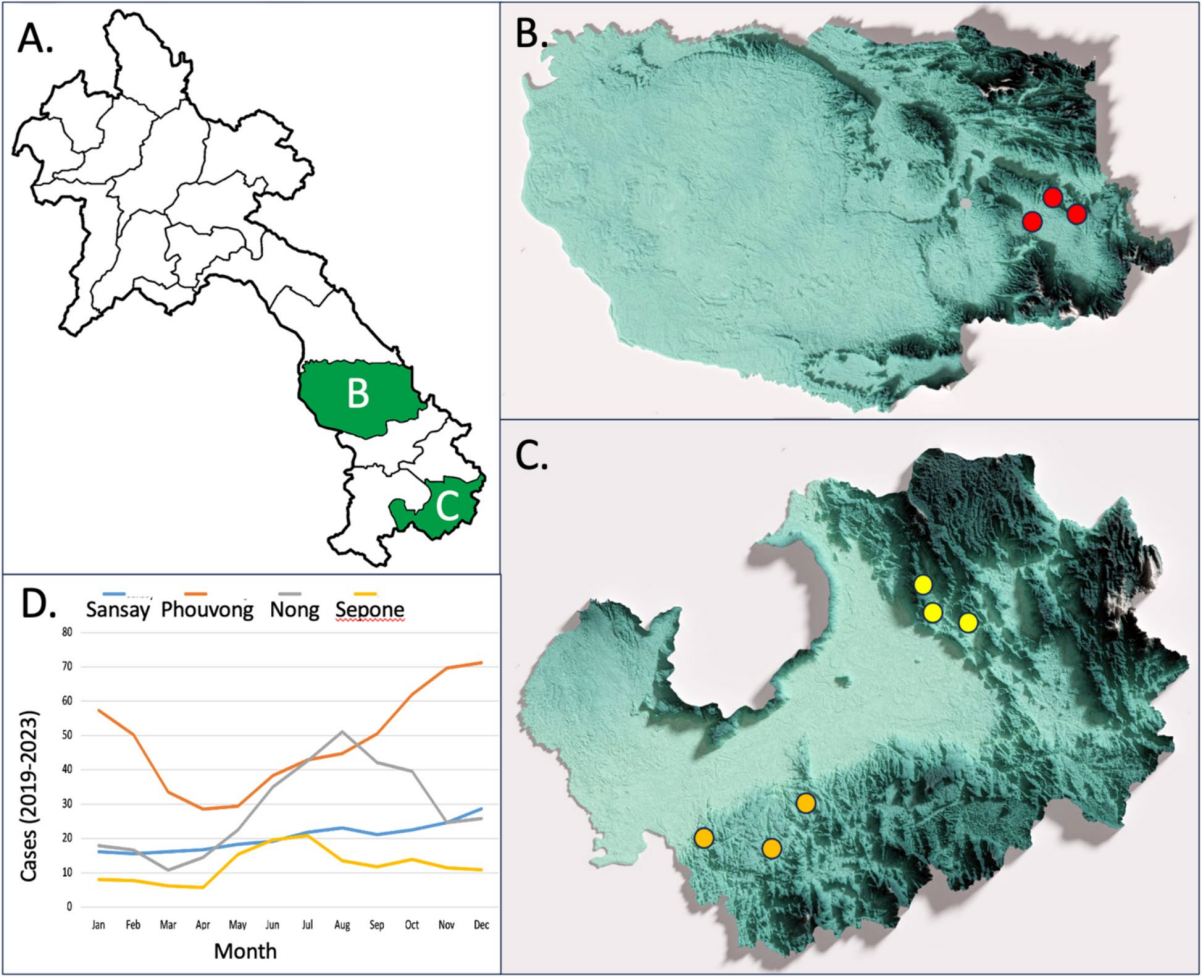
**A** The map of Lao PDR with collection provinces **B** Savannakhet and **C** Attapeu Provinces filled in. **B** Savannakhet province. Village sites in Nong and Sepone Districts, (red). **C** Attapeu Province. Cultivation sites (yellow) in Sansay district, and forest sites (orange) in Phouvong district. **D** Malaria cases reported by district between 2019 and 2023 (three month moving average). Topographical images from https://kongphaly.la/ Images created from NASA SRTM data and rendered by Blender

Data were collected from nine sampling sites across three different ecotypes: three sites in the village ecotype, three sites in the agricultural cultivation ecotype, and three sites in the interior forest ecotype (Fig. 1A–C). These sites were selected based on epidemiological data, its distinct ecotype (cultivation, forest, and village sites), and patient interviews (outlining exposure profiles) and logistical considerations. Cases with no travel history were being reported from Savannakhet, indicating potential village transmission, while in Attapeu almost all cases had a recent travel history to cultivation and/or forest locations outside the village (CMPE internal report). Sites were selected based on high number of malaria cases from the most recent monthly epidemiology data, on the presence of high-risk groups, and the presence or absence of interventions including LLINs, IRS and, the potential for long-lasting insecticide-treated hammock net (LLIHN) use. The agricultural cultivation sites included villages Phousay (15.000899; 107.077874), Tadseng (15.027147; 106.970694), and Moon (15.063165; 106.962187) in Sansay District, Attapeu Province. The Forest sites included villages Lamong (14.439439; 106.854679), Vonglakhone (14.613279; 106.657561), and Palia (14.649846; 106.879413) in Phouvong District, Attapeu Province. The village sites included Ponam (16.474071; 106.339004) and Houp (16.550097; 106.495882) in Nong District, and Palai (16.558271; 106.377787) in Sepone District, Savannakhet Province. LLINs are distributed routinely to all villages in Nong, Sepone, Phouvong, and Sansay districts as part of mass distribution campaigns every three years. IRS is only conducted as an ad hoc activity as part of a malaria outbreak response. The peak malaria transmission season in Sepone and Nong District starts in May, while in Phouvong and Sansay districts, peak transmission season runs from September through approximately January (Fig. 1D).

### Mosquito collections

Entomological surveillance was implemented during the rainy season 2022 (July to September) and the dry season 2023 (February to April). The operations were led by the CMPE entomological team with cooperation of provincial and district staff in each selected area. Indoor and outdoor (where applicable) HLCs were carried out in three sentinel structures for three days for each sampling period, from 18h00 to 06h00 in the village and cultivation sites, and for 48 h continuously in the forest sites. Indoor resting collections (IRC) were also conducted in five (non-HLC) houses per site, between 06h00 to 08h00, following the National Vector Surveillance guideline developed by the CMPE and WHO [20, 21]. The human biting rates were calculated as the mean number of mosquitoes landing per person per hour in each location (indoor/outdoor) by site.

#### Morphological mosquito identification

The morning following the collections, mosquitoes from both HLCs and IRCs were morphologically identified to the *Anopheles* genus and to species or group/complex in a field laboratory, using microscopes and appropriate identification keys for Southeast Asian *Anopheles* [22]. Identification was carried out on site by qualified entomologists from the CMPE.

#### Human behaviour observations (HBOs) and analysis

HBOs (8) were conducted alongside HLCs at the three HLC households and at the five IRC households in the three villages of Savannakhet (only in the rainy season), and the three cultivation sites of Attapeu (rainy and dry seasons). At the end of each HLC collection hour, The HLC supervisor documented the number of people inside and outside the sampling structure, the number of people awake and asleep, and the number of people using an LLIN. Data was verified and entered into an Excel spreadsheet.

Human behaviour observation-adjusted (HBO-adjusted) HBRs were calculated based on Monroe et al. [8], Martin et al. [9] and Finda et al. [12]. Briefly, site-based HBRs were multiplied by the proportion of observed individuals exhibiting different behaviour at each location/hour towards understanding spatial and temporal community exposure profiles.

### Epidemiology

Epidemiological data were extracted from the national malaria database: District Health Information System, Version 2 (DHIS2). Population data were estimated by using village-level data collected as part of the LLIN distribution campaign in 2019 and assuming an annual population growth rate of 1.4% (https://data.worldbank.org/country/lao-pdr). Annual parasite incidence (API) was calculated as the annual number of cases reported divided by the annual population, multiplied by 1000. Monthly parasite incidence was calculated as the number of cases reported per year, divided by the annual population, divided by 12, and multiplied by 1000. API and monthly parasite incidence were calculated by village. The 3-month moving average of the monthly parasite incidence was calculated by summing the incidence for a specific month with the preceding and subsequent months and dividing it by three. The 3-month moving average of the monthly parasite incidence between 2019 and 2023 for each village were displayed in a line graph with the HBR at the time of the entomological survey overlaid (Supplementary Table S1 and Supplementary Fig. S1 and Fig. S2).

## Results

### *Anopheles* species composition

A total of 1,012 *Anopheles* mosquitoes representing 12 morphologically identified species were collected in the three ecotypes using HLCs (Table 1). The raw data demonstrates that 56% of *Anopheles* were collected during the rainy season against 44% in the dry season. Approximately 20%, 50% and 30% of all *Anopheles* mosquitoes sampled were collected in the forest, cultivation sites, and villages respectively.

**Table 1:**
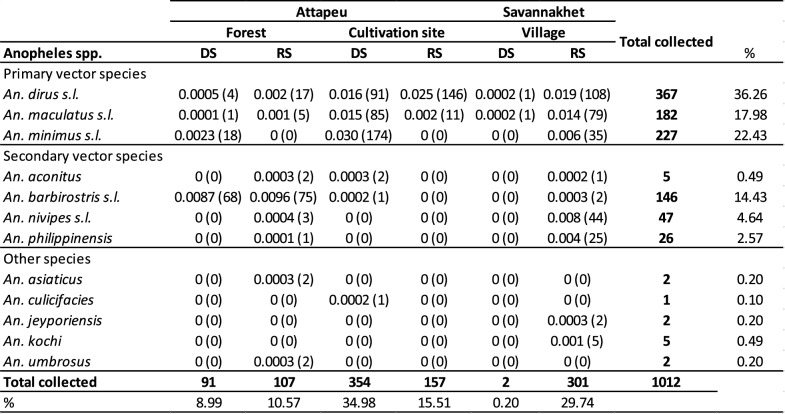
Species specific landing rates

Landing rates (a proxy for biting rates) for morphologically identified specimens collected by HLCs (combined results for both indoor and outdoor collections for the village site) during the dry (DS) and rainy season (RS) in the forest, cultivation sites and villages in Attapeu and Savannakhet over the study period. Biting rates (bites per person per hour) are followed by the number of mosquitoes collected in brackets

In village sites, HLCs were able to sample mosquitoes only in the rainy season. The most abundant species captured, almost all in the rainy season, included *Anopheles dirus *sensu lato (s.l.) (henceforth *An. dirus*) (n = 108, 35.8% of *Anopheles* in this ecotype), *Anopheles maculatus* s.l. (henceforth *An. maculatus*) (n = 79, 26.5%), *Anopheles nivipes* (n = 44, 14.6%) and *Anopheles minimus* s.l. (henceforth *An. minimus*) (n = 35, 11.6%). In cultivation sites, the most abundant species captured were *An. dirus* (n = 237, 36.4% of *Anopheles* in this ecotype), *An. minimus* (n = 174, 34%) and *An. maculatus* (n = 96, 18.8%). Interestingly all the *An. minimus* and most of the *An. maculatus* were caught in the dry season while more *An. dirus* were caught in the rainy season. In forest sites, the most abundant species captured were *Anopheles barbirostris* (n = 143, 72.2%), followed by *An. dirus* (n = 21, 10.6%). Other species captured, that represented less than 10% of the total collection by site are reported in Table 1.

### Anopheles bionomics: host-seeking behaviour

#### HBR in the three ecotypes

During the rainy season 2022, *An. maculatus* and *An. dirus* were the only primary species found in the three ecotypes, and *An. dirus* had the highest HBRs (> 0.01 bite per hour (bph)) in the cultivation sites and in the villages (Table 1). *Anopheles maculatus* had the highest HBR in the villages. *An. barbirostris* showed very high HBR in the forest in both seasons (> 0.087 bph). The HBRs in the villages were also high for *An. maculatus*, *An. minimus*, *An. nivipes,* and *Anopheles philippinensis*. For most of the species, HBRs were quite similar indoors and outdoors except for *An. dirus* which demonstrated greater exophagy. There were no mosquitos sampled during the dry season at the village sites. During the dry season of 2023, *An. maculatus*, *An. minimus* and *An. dirus* had the highest HBRs in the cultivation sites (Table 1). Again, *An. barbirostris* showed a very high HBR in the forest followed by *An. minimus*. Compared to the rainy season, the human population was not exposed to any mosquito bites in the villages in the dry season at the time of collections.

### Biting time in the villages (rainy season only, 2022)

**Fig. 2:**
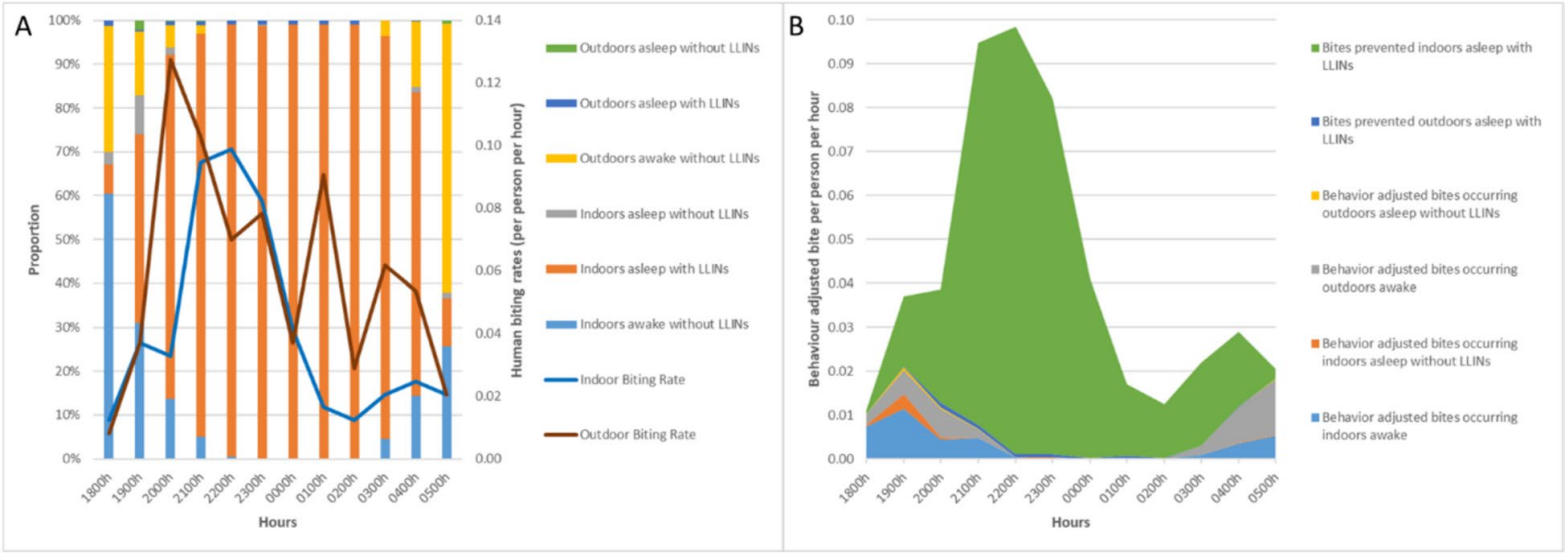
Village sites. *Anopheles* and human behaviour. **A** Directly observed *Anopheles* biting rates (based on HLCs) are outlined in dark red (outdoor) and blue (indoor) throughout the night while spatial and temporal presence, alongside intervention usage is in bar form. **B** Human behaviour-adjusted biting rates in three villages in Savannakhet, including bites prevented by LLINs (green)

Figure 2A depicts the biting times indoors and outdoors in the three villages in Savannakhet. No mosquitoes were collected in the dry season in the villages. The same behaviour were seen for each species across sites and are represented in one figure. For both indoors and outdoors collections, the results show that *Anopheles* were active throughout the night particularly for primary vectors *An. dirus* and, *An. maculatus.* The directly observed host-seeking, as characterized by HLCs, was documented throughout the night with an indoor peak between 21h00 and 24h00 indoors (Fig. 2A) and a regular activity of *Anopheles* throughout the night outdoors with a higher peak between 20h00 and 02h00 (Fig. 2A). *Anopheles dirus*, *An. maculatus*, *An. nivipes* and *An. philippinensis* were captured continuously between 20h00 and 06h00 both indoors and outdoors (Fig. S3A and S3B).

### Biting time in the cultivation sites

**Fig. 3:**
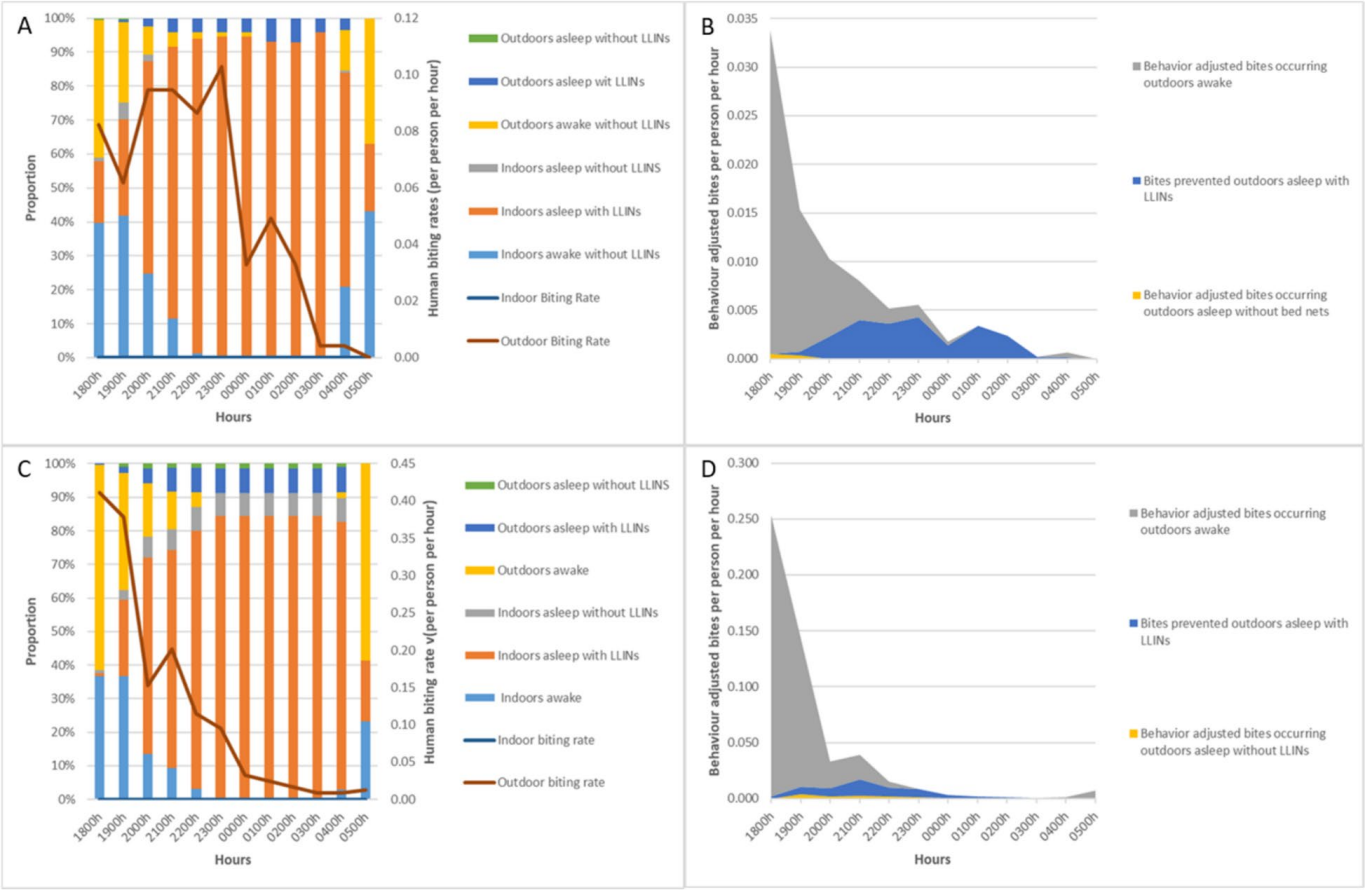
Cultivation sites. *Anopheles* and human behaviour. Directly observed *Anopheles* biting rates (based on HLCs) are outlined in dark red (outdoor) throughout the night while spatial and temporal presence, alongside intervention usage is in bar form—**A** Rainy season, and **C** Dry season. Human behaviour-adjusted biting rates are presented in (**B**). Rainy season, and **D** Dry season—both indicating primarily outdoor exposure

Figure 3A and C show the biting time of all *Anopheles* species collected between 18h00 and 06h00 in the CS sites in Attapeu. In the cultivation sites, the mosquitoes were only captured outdoors. During the dry season, *An. dirus* is the most predominant vector captured and is active throughout the night (Fig. S3C) while in the rainy season, the three major vectors *An. dirus*, *An. maculatus* and *An. minimus* have biting activities very high at dawn and reduce during the night (Fig. S3D).

### Biting times in the forest

Figs S3E and S3F and shows the biting time of all the *Anopheles* species collected over 48 h collections in the forest in Attapeu province in the rainy and dry seasons. The secondary vector *An. barbirostris* was collected almost every hour in the forest on human with a lowest number in the afternoon. The other important vector *An. dirus* was mostly collected during the night except few specimens captured at 09h00 in the rainy season and was less abundant in the dry season. *Anopheles minimus* was more abundant during the night in the dry season.

### Anopheles bionomics: indoor resting behaviour

Indoor resting collections were conducted following the SOPs of the Lao entomology surveillance guideline [21] in all the villages around 08h00 for two hours after the HLCs in the five houses dedicated for the indoor resting behaviour houses. No mosquitoes were seen or captured resting on walls, LLINs or other surfaces within the 24 houses visited.

### Human behaviour adjusted biting rates

#### HBO-adjusted bites in the villages (only in the rainy season, 2022)

Human behaviour related to location (inside or outside), sleep, and LLIN use (people asleep under LLINs or not) were characterized alongside HLCs (Fig. 2A). The proportion of people indoors increased between 18h00 and 22h00. Most people were asleep from 21h00 to 05h00. Directly observed *Anopheles* biting rates were adjusted to factor in human presence (inside or outside), time inhabitants went to sleep, and LLIN usage (Fig. 2B) [8, 12]. The very high use of LLINs observed in the three villages prevented approximately 42% of the total *Anopheles* bites, and 83% of *Anopheles* bites indoors. Gaps in protection were identified indoors and outdoors, in similar proportions, in the early evening and early morning.

### HBO-adjusted bites in the cultivation sites

During the rainy season, more than 60% of people were indoors in the cultivation sites (CS) at 18h00 and increased to 75% by 19h00 (Fig. 3A). Approximately 5% of the population from the CS sleep outdoors at night with a LLIN. Overall, most people are asleep between 21h00 and 05h00. About 40% of people were active outdoors at 05h00. The HBRs outdoors were the highest at 20h00 and slowly decreased throughout the night with a HBR of 0.02 bph at 05h00. The use of LLINS outdoors in the CS prevented approximately 26% of the total *Anopheles* bites (Fig. 3B).

During the dry season, the proportion of people indoors was already more than 60% at 20h00 and increased up to 85% at 21h00 (Fig. 3C). Figure 3C shows that in average, 6% of the population at the CS at night stay outdoors asleep with bed nets. Globally, most people are asleep between 20h00 and 04h00. From 19h00 onwards, a large proportion of people were indoors (60%) while at 05h00 about 60% of people were already active outdoors.

The use of LLINS observed outdoors in the CS prevented approximately 10% of the total *Anopheles* bites (Fig. 3D). Again, no mosquitoes were collected indoors. The HBRs outdoors were at 0.41 bite per person per hour at 6 pm, then decreased throughout the night with a HBR of 0.05 after midnight.

## Discussion

In the final push towards malaria elimination, it is critical for Laos to maximize the impact of available and limited resources. To this end, the integration of human and vector behaviour data using the ESPT (https://shrinkingthemalariamap.org/tool/entomological-surveillance-planning-tool-espt) allowed for the estimation of the remaining spatial and temporal gaps in protection in the context of existing vector control tools. Outcomes are discussed in terms of academic knowledge gains and remaining gaps, as well as operational decision-making and strategy-based recommendations moving forward. The timing and duration of these operationally based entomological collections were designed to reflect ecotype-based transmission dynamics. In both village and cultivation sites, people work during the day and sleep at night, mirroring mosquito activity patterns. As a result, exposure risk typically spans crepuscular to dawn periods (18:00–06:00 h). However, in forested sites with dense canopies, low-light conditions allow *Anopheles* mosquitoes to bite throughout both day and night [17, 23–26]. Additionally, forest-exposed populations often engage in both nighttime and daytime activities, increasing the potential for continuous exposure. Consequently, entomological sampling frames were optimized to represent these specific human and vector bionomic traits—including 48-h collections in the forest sites spanning day and night.

Overall, 12 *Anopheles* were identified to species, with seven species classified as malaria vectors. However, the identification of these species—with primary vectors being *An. dirus*, *An. maculatus* and *An. minimus*, were based on morphology, and may not reflect the primary vector status as sibling species included may not be vectors [27]. There might also be unknown and novel species included in these identifications as seen in other forested sites in the region [28].

Site and seasonal difference were seen with *Anopheles* species and densities. Interestingly, similar numbers of *Anopheles* were caught in the rainy and dry season reflected in the human landing rates with slightly more in the rainy season with species specific difference (Table 1). Higher densities may be attributed to the availability of more larval sites in the rainy season, and the presence of year-round larval sites may support consistent populations of *Anopheles* documented. The higher rainy season *Anopheles* densities agree with the epidemiological data that show higher case numbers in the rainy season (Fig. S1 and S2).

The sustained HBRs in the dry season (especially for *An. maculatus* and *An. minimus* in the cultivation sites, and for *An. minimus* in the forest) point to possible transmission in this season at these locations. The lower malaria reported in the dry season—inconsistent with the high *Anopheles* biting rates may be explained by the low sample size with not enough longitudinal entomological data, or seasonal species-specific vectorial capacity resulting in differing proportions of infectious bites, as well as human behaviour differences that map to differing exposure profiles. A larger temporal sampling framework alongside molecular identifications to specific species may allow for understanding how species-specific bionomic traits map to exposure profiles and disease incidence.

Another interesting outcome was data that demonstrates year-round and day time biting in the forest settings (Figs S3E and S3F). Biting rates in both seasons were sustained by *An. dirus* and *An. barbirostis* with the addition of *An. minimus* in the dry season and *An. maculatus* in the rainy season. This unusual but documented phenomenon [17, 23–26, 29–31], thought to occur due to the forest canopy (crepuscular-like, darker conditions), suggests both day and night exposure to infectious bites and the requirement for interventions that would be effective based on human behaviour in these periods.

At the village sites, *Anopheles* were only present in the rainy season with higher species diversity compared to other sites. The absence of *Anopheles* in the three villages in the dry season (same houses selected as 2022, rainy season) may be explained by weather being “very cold and very windy” at the period of collection (as reported by the CMPE staff conducting the study).

Spatial and temporal exposure to *Anopheles* bites (reflected in the epidemiological data) were based on ecotype and seasonal interactions between human behaviour, *Anopheles* behaviour and bed net usage. Overall, the major gaps in protection were outdoors in every ecotype with timing of exposure dependent on the specific transmission system: all day in the forest, early evening in the CS, and early evening and early morning at village sites. This was supported by entomological data in Savanakhet where transmission continues to occur in the villages (with six *Anopheles* species present), Attapeu (CS) with a high prevalence of the important vector *An. dirus* [13, 32], and in the forest with the presence of *An. dirus* and *An. barbirostris*. Though no mosquitoes were collected indoors, the use of LLIN indoors is an important protective measure in the CS with over 80% of the people using them during sleep hours.

A high proportion of *An. barbirostris* was collected in the forest in Attapeu. This species is widely distributed in Laos [33, 34] and Thailand and more globally in the Asian region [35]. Species in this complex have been reported as a vector of *P. falciparum* and *P. vivax* in Sri Lanka, Bangladesh, Indonesia, Timor Leste, as well as a secondary vector on the island of Borneo and as putative malaria vector in the southeastern Thailand [36]. Documentation of this vector complex in high densities in the forest points to two priority needs: a) an immediate malaria elimination-based need to appropriately target forest goers with appropriate tools (Table 2), and, b) research to evaluate the contributions of this species to malaria transmission in these ecotypes and high-risk populations.

**Table 2:** Intervention potential according to the environmental settings to reducing gaps in protection based on data generated in this study

Setting	Evidence based gaps in protection	Intervention potential as a primary or supplemental intervention based on data generated in this study					
		LLINs	Hammock LLINs	IRS	Zooprophylaxis	Spatial Repellents (Indoors/ outdoors)	Protective Clothing
Village	Early evening Early morning Outdoor exposure	Protective—continue	No	No	Possible, Requires evaluation	Potential indoors—evaluations towards scaleup required; Possible outdoors-evaluations required	Not recommended
Cultivation sites (CS)	Early evening Outdoor exposure	Protective, if used outside	Possible	No	Unlikely to be effective		Not recommended
Forest	24 h exposure Outdoors	No	Protective—continue	No	Not recommended		Possible

Adjusted HBRs indicate that people living in rural/forested areas in Attapeu and Savannakhet are exposed to *Anopheles* throughout the night. The use of LLINs remains important, but innovative tools and new strategies are needed to address remaining gaps in protection – outside and in the early evening/morning. The high use of LLINS observed in the three villages prevented approximately 42% of *Anopheles* bites. Similar results were observed in other studies [9, 37], where an analysis of exophagic vectors with human behaviour indicated a clear gap in protection even with high LLIN coverage. Though LLINs provide both personal and community protection [38], this intervention may be insufficient by itself in areas with early, outdoor-biting *Anopheles* [8, 12]. These results emphasize the need for new tools, as well as new research that evaluates novel paradigms directed at residual transmission occurring outside the functional scope of present interventions, accelerating progress towards elimination. Linking epidemiological and entomological surveillance data with human behaviour is important to identify gaps in protection, while further contextualizing new paradigms with environmental, social and cultural factors in remaining transmission areas in the southern parts of Laos needs to be considered.

No mosquitoes were found resting in the houses in the morning suggesting that IRS may not be useful if mosquitoes do not rest indoors. The lack of mosquitoes resting indoors in the early morning be explained by kitchen-smoke associated mosquito repellence considering that the kitchen is often located indoors or very close to the house and villagers start cooking around 05h00. Since sampling to evaluate indoor resting were based on morning collections only, it is not clear if there is any indoor resting behaviour exhibited earlier in the night as vectors may enter, feed, rest and leave before sampling was conducted [39]. Further studies that evaluate potential indoor resting throughout the night are needed in order to more clearly determine the appropriateness of IRS and its efficacy in the remaining transmission areas in Laos.

Significant biting occurred at forest and cultivation sites with data demonstrating exposure not just throughout the night but also during the daytime in the forest. Since *Anopheles* sampling was only conducted during the night in CSs (18h00 to 06h00), and high biting was seen at the start of sampling at 18h00, extending these collections into the day may further reveal exposure spaces and times. Exposure patterns in the CSs (Fig. 3) suggest that the increased use of LLINs or LLIHNs may potentially protect people against 48% of *Anopheles* bites. Furthermore, it would be useful to know if there is a difference in the indicators depending on the type of culture (rice, cassava, and other crop sites). Observations from data collectors suggest that updating the HBO methodologies to include additional drivers of protection and exposure, such as LLIHNs use, use of repellents, as well as food harvesting and hunting activities, may further outline the interactions of mosquito and humans and interventions in these targeted spaces. Towards this, a qualitative data on routine human practices at the forest and cultivation sites that includes important data on spatial and temporal presence, specific human activities related to exposure, types of crops cultivated related to vector densities and human behaviour, protective methods used, and other possible drivers of transmission or protection, is planned as part of an ethnographic study in higher malaria incidence areas in Laos.

Evidence generated demonstrated that most exposure to *Anopheles* bites and therefore residual transmission is occurring outside, with timing dependent on the ecotype and season. In villages, outdoor exposure occurred in the early evening and morning hours of the rainy season; in cultivation sites, outdoor exposure occurred throughout the year in the early evening hours; in the forest, outdoor exposure occurred at all hours of the day and night. To address these gaps in protection, several vector control tools may be appropriate to pilot and evaluate (Table 2).

## Conclusion

In Laos, understanding where and how transmission is persisting, monitoring trends, and guaranteeing appropriate and effective vector control alongside epidemiological interventions, is critical during this last phase of malaria elimination. This study has generated actionable evidence that outlines vector, human, and season-based drivers of gaps in protection as well as intervention effectiveness in the forest, cultivation areas and in remaining villages with high malaria transmission. Generated data suggests that a more thorough evaluation of parasite transmission within forest goers and agricultural workers and their behaviour would provide important data towards more effective vector control necessary for malaria elimination. LLIN use remains very important to prevent most indoor exposure, but the spatio-temporal overlap between vector-biting behaviour and human behaviour outdoors demonstrates that additional approaches are required to reduce residual malaria transmission and to achieve elimination. Strengthening capacity for integrated data analysis will also be a critical part of the national program’s preparations for post elimination and the prevention of reintroduction. In elimination and post elimination settings, entomological surveillance will often be triggered by the detection of a single case, and data generated in this study will be vital to determine receptivity and transmission risk, as well as appropriate responses required, including vector control interventions.

## Supplementary Information

Supplementary Material 1: Table S1. Epidemiological data by village and transmission period in 2 provinces in Laos 2019-2023. Figure S1. Monthly parasite incidence by village, 2019-2023. Figure S2. Monthly parasite incidence by year and village, 2019-2023.

Supplementary Material 2: Figure S3. Biting times on human in the villages, cultivation sites and in the forest by *Anopheles* mosquitoes, collected by sites, seasons, and indoor/outdoor.

## Data Availability

No datasets were generated or analysed during the current study.

## Abbreviations

BPH: Bite per human
CMPE: Centre for Malaria, Parasitology and Entomology
CS: Cultivation sites
DHIS2: District Health Information System, Version 2
HLC: Human Landing Catching
HBR: Human biting rates
HBO: Human behaviour observations
HRPs: High-risk populations
IPTf: Intermittent preventive treatment for forest goers
IRC: Indoor resting collections
IRS: Indoor residual spraying
LLIHNs: Long-lasting insecticidal hammock nets
LLINs: Long lasting insecticide treated nets
WHO: World Health Organization
