# Supplementary material for: Linking Anopheles bionomics and human behaviour in the Lao PDR
Source: Malar J. 2025 Jul 2;24:213. doi: 10.1186/s12936-025-05435-1 (PMC12225035; doi:10.1186/s12936-025-05435-1)
Supplement: Supplementary file 1 — Supplementary Material 1: Table S1. Epidemiological data by village and transmission period in 2 provinces in Laos 2019-2023. Figure S1. Monthly parasite incidence by village, 2019-2023. Figure S2. Monthly parasite incidence by year and village, 2019-2023. [file 12936_2025_5435_MOESM1_ESM.pdf]

Supplementary Table 1. Epidemiological data by village and transmission period in 2 provinces in Laos 2019-2023.

Province	District	Village	Date of survey	Season	HBR	Population 2023	Number of cases					Annual Parasite Incidence				
							2019	2020	2021	2022	2023	2019	2020	2021	2022	2023
Attapeu	Sansay	Phousay	31 March to 2 April 2023	Dry	0.28	1148	10	13	38	50	0	9	12	35	45	0
		Phousay	1-3 September 2022	Rainy	1.37											
		Tadseng	26-28 March 2023	Dry	2.91	361	109	133	117	13	0	325	391	339	37	0
		Tadseng	6-8 September 2022	Rainy	0.56											
		Moon	22-24 March 2023	Dry	3.33	512	47	179	85	13	0	99	370	173	26	0
		Moon	11-13 September 2022	Rainy	0.98											
	Phouvong	Lamong	29-30 March 2023	Dry	1.92	491	29	85	21	15	0	63	183	45	31	0
		Lamong	1-2 September 2022	Rainy	5.92											
		Vonglakhone	26-27 March 2023	Dry	2.92	595	19	5	55	101	0	34	9	97	175	0
		Vonglakhone	5-6 September 2022	Rainy	2.00											
		Palai2	22-24 March 2023	Dry	2.75	684	93	46	134	47	0	146	71	205	70	0
		Palai2	9-10 September 2022	Rainy	1.00											
Savannakhet	Nong	Ponam	21- 23 February 2023	Dry	0.00	222	9	7	86	3	0	43	33	404	14	0
		Ponam	27-29 July 2022	Rainy	2.85											
		Houp	30-1 August 2022	Dry	1.43	293	79	7	55	16	0	290	25	196	56	0
		Houp	22-24 February 2023	Rainy	0.00											
	Sepon	Palai1	25-27 February 2023	Dry	0.00	449	66	129	148	0	0	158	304	344	0	0
		Palai1	1-3 August 2022	Rainy	1.17											

Supplementary Figure 1 demonstrates the disruption of the natural epidemiological trends following the introduction of the Accelerator Strategies, which were intensified interventions to accelerate malaria elimination, and specifically targeted drug administration (TDA) for malaria elimination were implemented in the target communities in between March and October 2022 and May to September 2023. The impact was particularly evident in 2023 with low monthly parasite incidence across all villages and months. Due to the change in epidemiology, associations between HBR and API are challenging to infer. To overcome this, the monthly parasite incidence from previous years was used as an indicator of the expected monthly parasite incidence in absence of such interventions (Supplementary Fig.2).

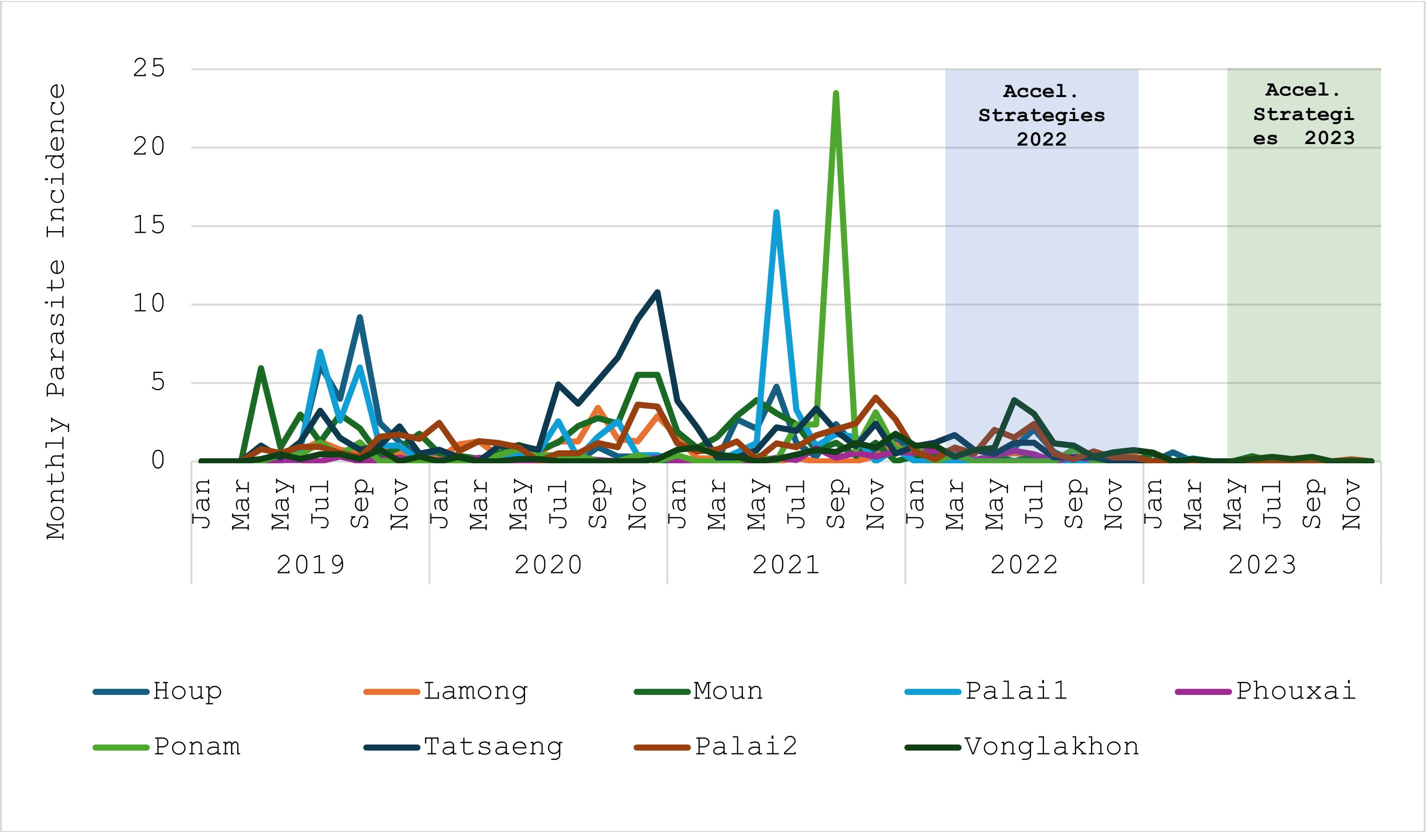

Supplementary Figure 1. Monthly parasite incidence by village, 2019-2023. Accel. Strategies: Accelerator Strategies were intensified interventions, specifically targeted drug administration and intermittent treatment for high-risk groups, implemented in the target communities between March and October 2022 and May to September 2023.

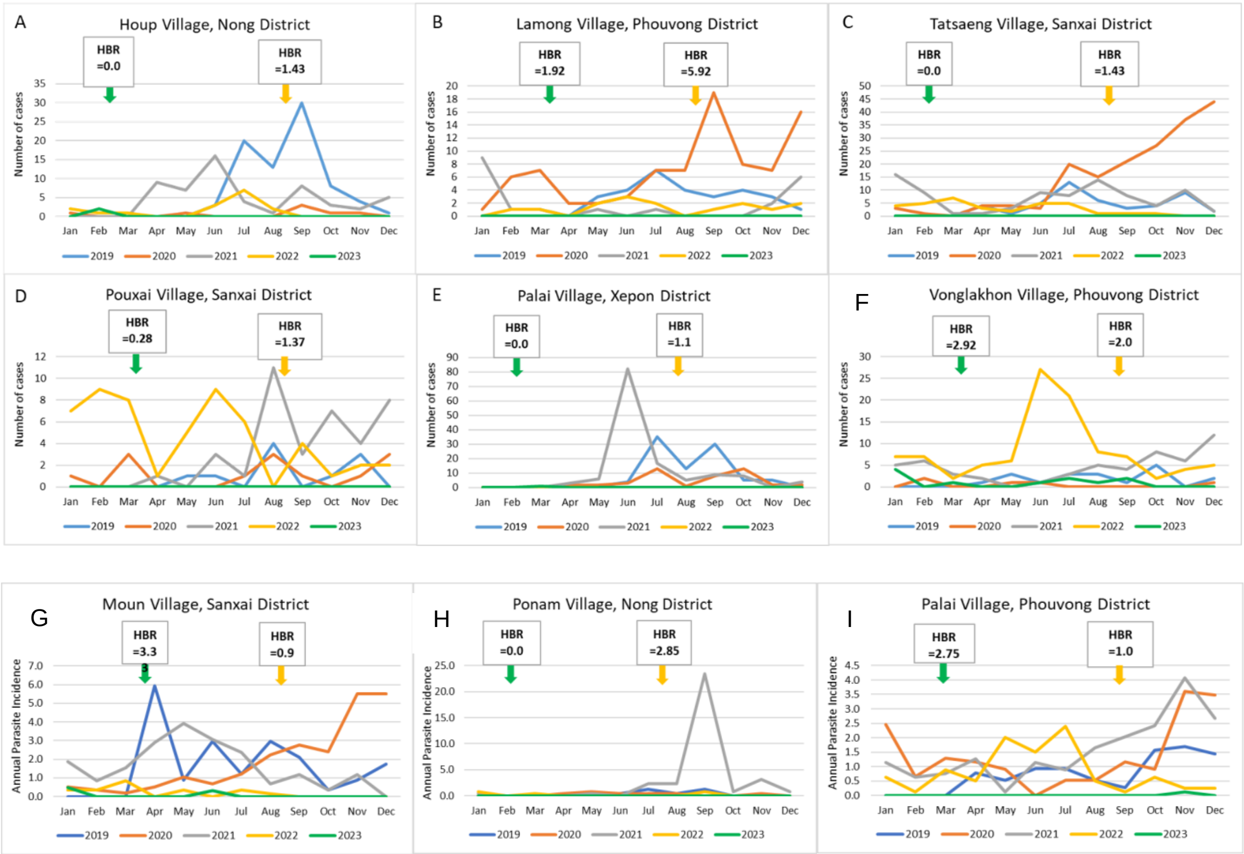

Supplementary Figure 2. Monthly parasite incidence (3-month moving average) by year and village, 2019-2023. HBR: human Biting Rates.
