# Supplementary material for: Linking Anopheles bionomics and human behaviour in the Lao PDR
Source: Malar J. 2025 Jul 2;24:213. doi: 10.1186/s12936-025-05435-1 (PMC12225035; doi:10.1186/s12936-025-05435-1)
Supplement: Supplementary file 2 — Supplementary Material 2: Figure S3. Biting times on human in the villages, cultivation sites and in the forest by Anopheles mosquitoes, collected by sites, seasons, and indoor/outdoor. [file 12936_2025_5435_MOESM2_ESM.pdf]

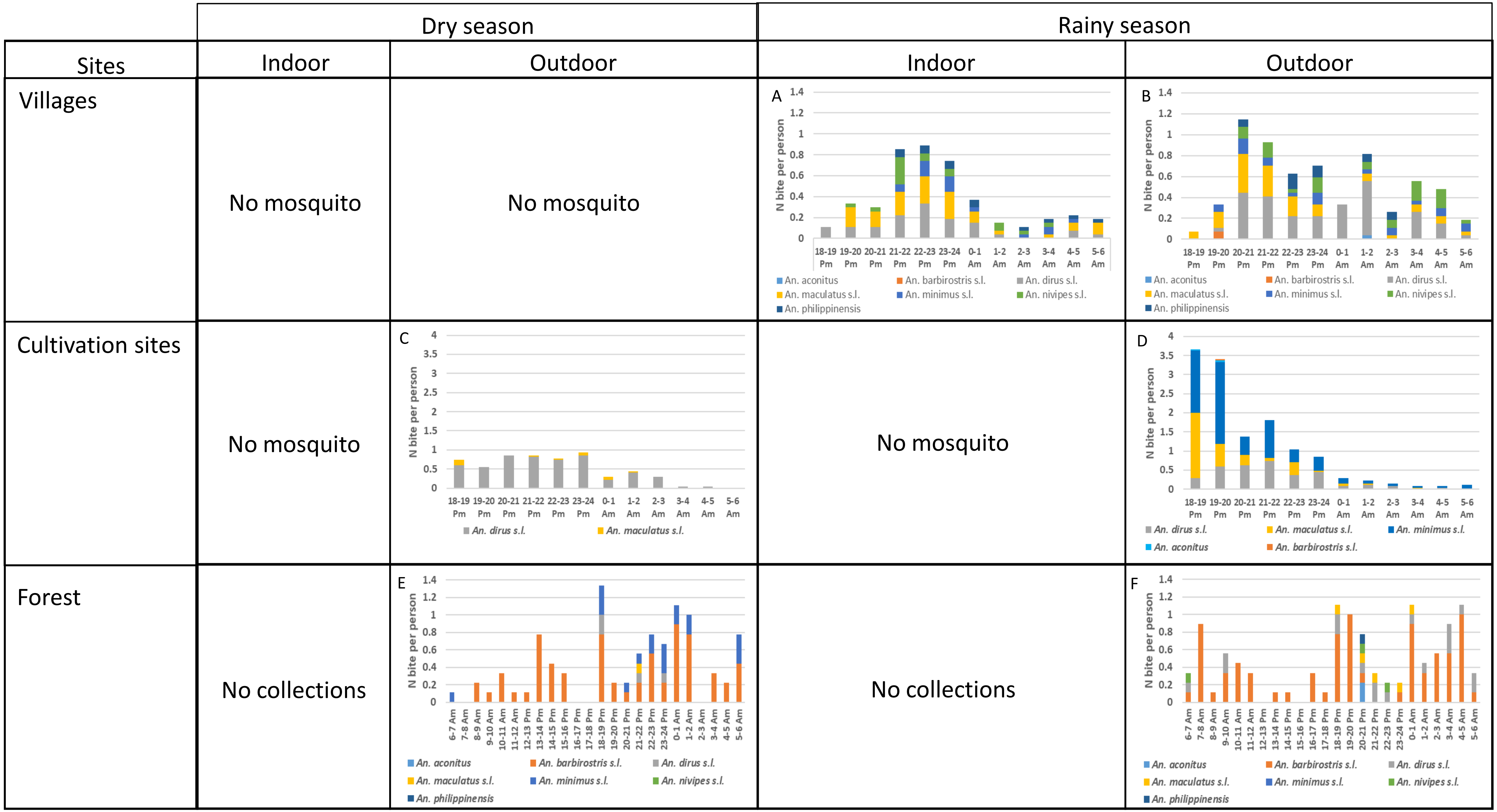

Supplementary figure S3. Biting times on human in the villages, cultivation sites and in the forest by *Anopheles* mosquitoes, collected by sites, seasons, and indoor/outdoor.
